# Gender differences and inflammation: an in vitro model of blood cells stimulation in prepubescent children

**DOI:** 10.1186/1476-9255-7-28

**Published:** 2010-06-02

**Authors:** Georges JA Casimir, Fabienne Heldenbergh, Laurence Hanssens, Sandra Mulier, Claudine Heinrichs, Nicolas Lefevre, Julie Désir, Francis Corazza, Jean Duchateau

**Affiliations:** 1 Department of Pulmonology and Allergology, Université Libre de Bruxelles (ULB), University Children's Hospital Queen Fabiola, Avenue J.J. Crocq 15, Brussels, 1020, Belgium; 2 Laboratory of Paediatrics, Université Libre de Bruxelles (ULB), University Children's Hospital Queen Fabiola, Avenue J.J. Crocq 15, Brussels, 1020, Belgium

## Abstract

**Background:**

Gender influences clinical presentations and markers in inflammatory diseases. In many chronic conditions, frequency of complications is greater in females, suggesting that continuous inflammatory reaction may induce greater damage in targeted organs and functions.

**Methods:**

To investigate gender dimorphism at a cellular level, we evaluated the production of cytokines implicated in inflammatory processes (IL -1, IL- 6, PGE-2 and TNF alpha), in healthy prepubescent children of both sex and Turner's syndrome (TS) patients (genotype XO). We used stimulation by LPS (0.2 and 1 ng/ml) and Pokeweed Mitogen (PWM) on overnight cultures from whole blood samples, collected in 57 subjects: 22 girls/26 boys (5-96 months), and 9 TS patients (6-15 years). The primary outcome was to evaluate if gender influences the production of cytokines, with potential relation to X chromosome monosomy. Secondary endpoints were to relate different cytokines level productions and conditions.

**Results:**

We confirm the male over female increased cytokine productions already observed in adults. This is contrasting with numerous observations obtained in vivo about increased production of inflammatory markers in females (CRP, ESR and neutrophil counts), as we recently reported in children. Relative variations of the dimorphism according to stimulus, its concentration and cytokine type are discussed, presenting IL6 with a modulating function that could be more potent in males. TS subjects follow mostly the male pattern of reactivity, sustaining the role of some gene expression differing with X chromosome monosomy and disomy.

**Conclusions:**

Persistence of the latter dimorphism throughout life casts doubts on its direct relationship with individual hormonal status, as already documented by others in vitro, and supports the need for alternative hypothesis, such as the influence of X chromosome gene products escaping X inactivation in females and absent in subjects with X monosomy (males, TS).

## Background

Inflammatory markers during acute inflammation as C-reactive protein (CRP), erythrocyte sedimentation rate (ESR) and neutrophil count (NC) are, as a mean, higher in female than in male children [1]. Gender also influences clinical presentations (higher mean duration of temperature under antibiotic administration and longer mean period of hospitalisation in females)

Gender differences are also evident in chronic inflammatory diseases: a higher median cumulative dose of systemic corticosteroids was needed to reverse wheezing in female children with severe asthma crisis. From 2 years of age, symptoms and inflammatory status are accentuated in females suffering from cystic fibrosis (CF), and in sickle cell anaemia, vasoocclusive crisis (VOC) occur more frequently in females [2]. In addition, in many chronic conditions and connective tissue diseases [3], frequency of complications is greater in females, suggesting that continuous inflammatory reaction may induce greater damage in targeted organs and functions.

Conversely, the prognosis is better for females than males during sepsis [4,5] or extended burns [6,7], which could reflect a more efficient mobilization of neutrophils and/or related inflammatory reaction.

One possible explanation is that inflammatory reactions are driven by the hormonal status. However, clinical data obtained before puberty implicates potential differences in gene expression depending on sexual chromosomes rather than hormonal status as the latter is largely immature and sexual hormones are far less abundant.

Attention has recently been drawn to some rare genes on the X chromosome that are involved in the inflammatory cascade [8-10]. As the normal silencing process of one of the X chromosomes is incomplete in females [reviewed in [11]], some inflammation related genes could therefore be over expressed compared to males and individuals with Turner syndrome, who lack the second X chromosome. Additionally, some other inflammation related genes are expressed on X [8-10] and sometimes also on Y chromosomes [12], allowing some undisclosed balance that could be important. Sexual dimorphism might be related to sex-specific downstream mechanisms in the cell signalling cascade. For this reason we have investigated blood cells from male and female prepubescent children, and from girls affected by Turner syndromes (who are natural examples of X chromosome monosomy).

Several publications have already reported the production of higher levels of cytokines by male's cells, ex vivo [13,14] in humans [15-18] and in animals [19-21].

We have explored the capacity of whole blood cells to produce several major cytokines involved in the generation and control of inflammation, in vivo.

Short term cultures of whole blood have been demonstrated as a valuable and low cost method to assess monocyte derived cytokine production [22].

We have selected a direct stimulation with graded doses of LPS and Pokeweed Mitogen lectin as stimulants in vitro.

LPS-induced signalling in macrophages, and in other LPS-responsive cells such as neutrophils, is known to be initiated by interaction of LPS with LPS-binding protein (an acute phase serum protein), followed by subsequent interaction with membrane-localized CD14, membrane-bound toll-like receptor (TLR) 4 and MD-2 [23,24]. This leads to the release of multiple inflammatory and anti-inflammatory mediators [24,25].

Recently a new model of LPS interaction has been proposed including a signalling complex of receptors, formed following LPS stimulation, which comprises heat-shock proteins (Hsps) 70 and 90, chemokine receptor 4 (CXCR4), growth differentiation factor 5 (GDF5) and later TLR4 [26]. Responses to different pathogens vary depending on cell type, composition of supramolecular activation clusters and intracellular adaptor molecules. According to this model, triggering receptor expressed on myeloid cells (TREM)-1 would be involved in the inflammatory response caused by bacteria on neutrophils and monocytes.

As an alternative, we used the lectin Pokeweed Mitogen, which is non specifically stimulating to white blood cell membranes, binding preferentially to monocytes rather than to T cells [27], and binding poorly, if at all, to neutrophils [28].

## Methods

### Subjects

A group of 57 healthy children (22 girls, 26 boys, and 9 Turner's syndrome patients, attending day surgery for tonsillectomy, circumcision, or strabismus) were enrolled for analysis of in vitro production of several cytokines induced by proinflammatory agents. In all cases, blood was examined for general anaesthetic purposes before surgery. Both girls and boys were prepubescent (between 5 and 96 months). Patients with Turner Syndrome were older (between 72 and 186 months). The study was approved by the Ethical Committee of the University Children's Hospital Queen Fabiola.

### Measurements of cytokine production in cell culture supernatant (IL-1, IL-6, PGE-2 and TNF alpha) after stimulation by LPS and PWM

Two millilitres of venous blood were collected on Calparine^® ^(25 μl of Calparine, 5000 UI of calcium heparinate, Sanofi-Pharma, 95 A23, Belgium 1831 Diegem) in sterile syringes. They were kept at room temperature and tested within 2 hours.

Blood was mixed in a 1/10 ratio with an RPMI 1640, buffered with bicarbonate and Hepes, containing L-glutamine medium (Gibco, BRL, Life Technologies LTD, Paisley, UK) and 0.5 ml of Geomycine^® ^(Schering-Plough, B^3^-H^2^, Uccle, Brussels) warmed at 37°C. All the instruments were endotoxin-free and non pyrogenic. After a 1-hour period of acclimatisation in a humidified incubator at 37°C in an atmosphere of 5% CO2 and 95% air mixture (Heraeus HBB 2472b, Heraeus Instrumente GmbH, Hanau, Germany), cell cultures were stimulated with LPS (LPS for culture, E. Coli, serotype 0111/B4, lot. 026. b26 Ref. L2654, Sigma Chemical Co., St Louis, Mo., USA) at a final concentration of 0.2 and 1 ng/ml and Pokeweed mitogen at 1/1000 (RPMI 1640). In control samples, the LPS volume was replaced by culture medium (RPMI 1640). Cell cultures were incubated for 16 h at 37°C as described above until the supernatant was collected after centrifugation (2,200 rpm for 5 min) and frozen at -70°C until assay.

TNF alpha, IL-6, IL-1 and PGE-2 were determined using an immunoenzymetric assay from Medgenix^®^, (Biosource, Fleurus, Belgium), according to the manufacturer's recommendations for culture supernatants. It is a solid- phase enzyme amplified sensitivity immunoassay performed on microtiter plates, based on the Oligoclonal System^® ^in which several monoclonal antibodies directed against distinct epitopes of the cytokines are used, allowing high sensitivity. Specificity of the assay has been controlled by the manufacturer by excluding cross reactivity towards 25 other cytokines, including growth factors. PGE-2 was also measured by immunoassay of SPI Bio (Cayman # 514016, Estonia, Tallinn). The minimal detectable concentration was 3 pg/ml for TNF alpha, 2 pg/ml for IL-6 and 2 pg/ml for IL-1.

### Statistical Analysis

The Mann-Whitney test was used for nonparametric variables. Multiple regression analysis was used to determine related variables, and the Kruskal-Wallis test was used to analyse relationships between multiple nonparametric variables, with the Bonferroni correction when necessary. For direct paired comparisons, paired Wilcoxon tests were used.

## Results

### 1. Cytokine productions by stimulation with LPS and PWM

#### IL-1 response

**Figure 1:**
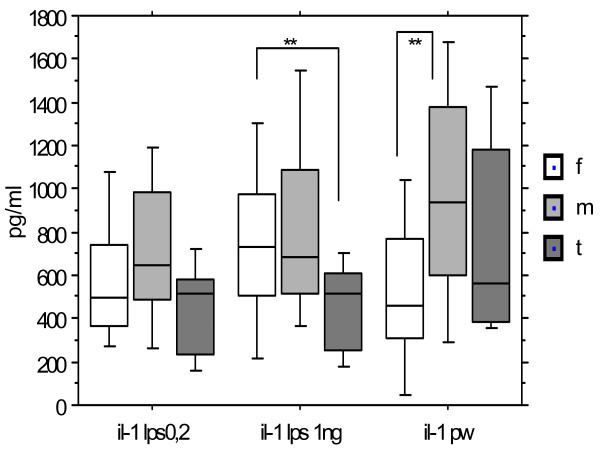
**IL-1 production after stimulation by LPS (0.2 and 1 ng/ml) and PW according to the gender**. Females (n = 19), males (n = 26), Turner syndrome patients (n = 9). Median, P 25-75, extremes. ** p < 0.03 between females and Turner after LPS 1 ng/ml. ** p < 0.01 between females and males after PW. p < 0.03 between females at 0.2 and 1 ng/ml. p < 0.02 between males at 0.2 and 1 ng/ml.

Figure 1 displays the variations of IL1 levels observed in 19 females, 26 males and 9 Turner syndrome subjects, with LPS at concentrations of 0.2 ng/ml and 1.0 ng/ml. The level distributions are widely overlapping and medians are not statistically different between groups except with the concentration of 1 ng/ml of LPS where girls from the Turner syndrome group had a significantly lower median production than females (p < 0.03).

Increasing the concentration of LPS increased the median production of IL1 in females and in males (p < 0.036; p < 0.02 respectively; paired Wilcoxon) but not in girls with Turner syndrome. The magnitude of observed changes in males and females were similar with a similar reactivity in response to LPS to produce IL1, while the Turner group has a lower reactivity.

Using a non CD14/Toll-like restricted stimulus as PWM, binding preferentially to monocyte membranes, males produced a higher median level of IL1 than females (p = 0.01). The median level for the Turner group was intermediate not statistically distinct from either of the other groups.

#### IL-6 response

**Figure 2:**
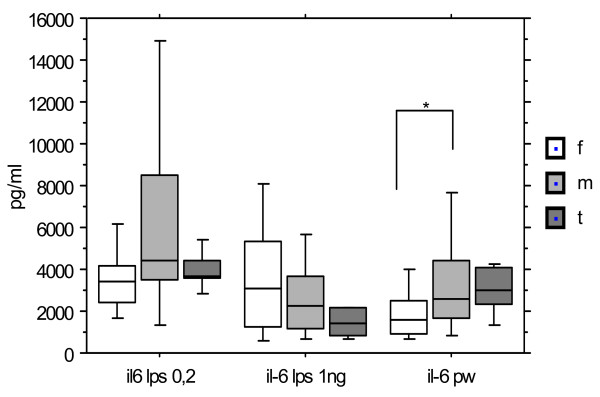
**IL-6 production after stimulation by LPS (0.2 and 1 ng/ml) and PW according to the gender**. Females (n = 19), males (n = 26), Turner syndrome patients (n = 9). Median, P 25-75, extremes. * p < 0.05 between males and females after PW. *** p < 0.005 between males at 0.2 and 1 ng/ml. *** p < 0.004 between Turner at 0.2 and 1 ng/ml.

Figure 2 displays the variations of IL-6 levels observed for the same groups in same conditions. Again, distributions are widely overlapping, and direct comparisons of medians do not differ statistically between groups for both concentrations of LPS.

Exploring the dose effect of LPS on individual IL6 production, a dramatic significant reduction was seen in males with increasing LPS concentration (p < 0.005), while in females, median levels were unaffected. Thus cellular reactivity to LPS for IL6 production differs between genders in terms of dose response. Moreover the dose effect in Turner syndrome group is equivalent to that of males as median IL6 production is also dampened with the higher LPS concentration of 1 ng/ml compared to 0.2 ng/ml (p < 0.004: paired Wilcoxon).

With PWM stimulation, males produced a higher median level of IL6 than females (p < 0.05), while Turner group produced a median level equivalent to the male group, but not statistically distinct from either group. Targeting by PWM, male monocytes produced more IL6 than females, at least at this relatively low level of stimulation. The level of PWM induced IL6 production is lower than that reached with the lowest concentration of LPS.

#### TNFα response

**Figure 3:**
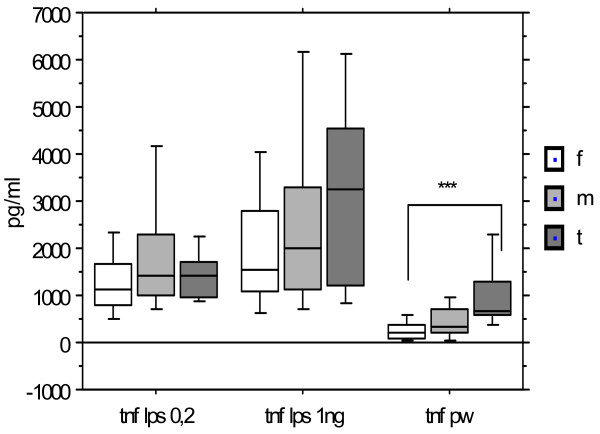
**TNFα production after stimulation by LPS (0.2 and 1 ng/ml) and PW according to the gender**. Females (n = 19), males (n = 26), Turner syndrome patients (n = 9). Median, P 25-75, extremes. *** p < 0.001 between Turner and females after PW.

Figure 3 displays the variations of *TNFα *levels observed for the same groups in same conditions. The distributions are widely overlapping; direct comparisons of medians do not differ statistically between groups for both LPS concentrations.

Increasing the concentration of LPS increased the median production of TNF *α *in females, in males and Turner group (p < 0.001; p < 0.05; p < 0.02; paired Wilcoxon). After PWM, females produced a lower median level of TNFα than Turner syndrome patients (p < 0.001), while median level in the male group was intermediate, and was not statistically distinct from either of the other groups.

#### PGE-2 response

Distributions in PGE-2 levels observed for the groups using LPS at 0.2 ng/ml are widely overlapping. Comparisons of medians showed greater PGE-2 production in males (median: 1324 and extremes: 317-2790) compared with females (median: 655 and extremes: 165-2468) which was statistically different (p < 0.02: Kruskal Wallis; Bonferroni protection). The Turner syndrome group showed an intermediate median (median: 934 and extremes: 112-2251) which did not differ statisticaly from the other groups.

### 2. Interrelationship between IL1 and IL6 productions on cells stimulation by LPS

**Figure 4:**
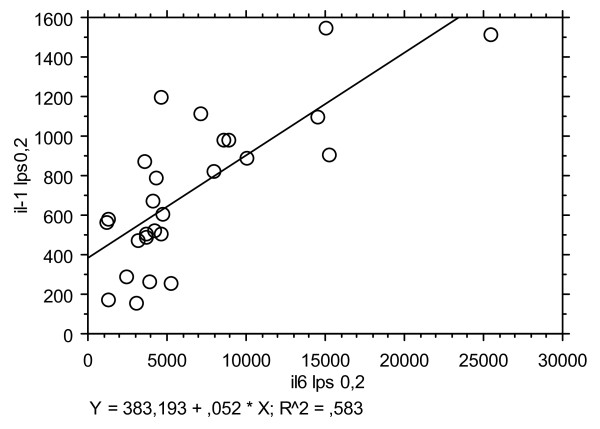
**Regression graph for IL-1 production according to IL-6 levels after stimulation by LPS 0.2 ng in males**. **** p < 0.0001 males.

Figure 4 illustrates the relationship between IL1 and IL6 production in vitro under stimulation with LPS at 0.2 ng/ml in males. A highly significant relationship was seen exclusively in the male group (R^2 ^= 0.58; linear regression; p < 0.0001) and not in females (R^2 ^= 0.009; p = 0.68) or in Turner patients (R^2 ^= 0.076; p = 0.36). No such relationship was observed for stimulations with higher concentrations of LPS, or with PWM.

### 3 Interrelationship between TNFα and IL6 productions on cells stimulation by LPS and PWM

TNFα level was proportional to that of IL6 in both gender groups, in a highly significant way, which was demonstrated by a linear regression analysis between the two cytokine levels for all the data related to some stimulation conditions, and in each separated group.

Table 1 summarises the data and their statistical significance for males and females.

**Table 1:** Relationship between TNF and IL6 productions according to gender in different stimulations

Stimulus		R^2^	slope	SD	p	dF
LPS 0.2 ng/ml	Male	0.62	0.15	0.024	<0.0001	23
	Female	0.49	0.25	0.057	<0.0004	19
	Diff. slopes*		- 0.10	z = -7.322	<0.0005	
LPS 1 ng/ml	no significative relations nor differences for male and female groups					
PWM	Male	0.26	0.06	0.02	<0.009	23
	Female	0.21	0.13	0.06	<0.04	19
	Diff. slopes*		- 0.07	z = -4.991	<0.0005	

*: T-test on slopes from male and female groups

The relationship between TNFα level and IL6 production was observed for PWM stimulation, LPS stimulation at the concentration of 0.2 ng/ml and not at 1 ng/ml.

Moreover, the degree of dependency, evaluated by the slope of the regression line, varied with the gender groups with a clearly significantly lower coefficient (slope) in males, meaning that the same increase of IL6 production would be associated with a lower increase of TNFα in males than in females.

## Discussion

We investigated gender dimorphism in healthy prepubescent children on the production of 3 cytokines (IL -1, IL-6, TNF alpha) and prostaglandin E2 implicated in inflammatory processes at the cellular level. This is the first report in this population; other studies have been performed in human adult subjects [16-18] and young animals.

It follows the documentation of such gender related dimorphism in vivo occurring in chronic, as well in acute inflammatory diseases [1,2,29-32]. Female children display higher production levels of inflammatory markers as well as suffering from prolonged fever periods and higher medication scores.

Our approach confirmed the generally observed trends of increased production of some cytokines in males compared with females. This situation contrasts with numerous clinical observations in vivo of increased production of inflammatory markers in females. The persistence of this dimorphism over the whole lifetime casts doubts on its direct relationship with the individual hormonal status. Previous reports of the literature showed a lack of 17 oestradiol or progesterone influence on LPS stimulated whole blood [33,34], whatever the plasma concentration or any experimental addition to the cultures. By the way, if our Turner subject group is older than our girls and boys groups, it is known that their sex hormonal status does not exceed that of normal prepubescent girls [35].

This is why an alternative hypothesis concerning genetic factors as permanent, life-long characteristics is attractive. We have therefore introduced the testing of girls with Turner syndrome (X0) as genotypic variants differing from females (XX) and males (XY) with the aim of identifying differences that could be related to X chromosome disomy and monosomy.

Several genes located on the X chromosome code for molecules involved in the inflammatory cascade [8-10]. Moreover, if allelic exclusion of one X chromosome occurs, 10-15% of genes from the silenced X chromosome escape this inhibition [8,9].

Our observations document that gender differences can depend on the type and intensity of the stimulus, and vary according to the considered cytokine.

LPS induced similar levels of IL1 production in males and females, with levels increasing in both with LPS concentration in the range used. The Turner group had no modification of IL1 with increased LPS concentrations. This highlighted a clear difference compared with females, and argues in favour of their lower reactivity to LPS.

The use of PWM induced a higher median level of IL1 in males compared with females, while levels in the Turner group appeared less pronounced (not statistically significant). It therefore appears that gender differences between our groups depend on the type and intensity of the stimulus addressing the cells.

The difference between LPS and PWM results could be related to different cell and receptor targeting as already mentioned.

Conversely, PWM binds almost exclusively to monocytes and not to neutrophils, to unspecified receptors. This allows some partition of cellular compartments in cytokine production, although reciprocal influence of monocyte-neutrophil mixtures cannot be ignored.

Considering IL6 production, it appeared that selected concentrations of LPS were in a plateau range of responses in females. In parallel experiments, males displayed a significantly increased IL6 production over females at 0.2 ng/ml LPS which was reduced at in vivo 1 ng/ml,. Therefore, the males displays an increased response to LPS compared to females, the Turner patients following a parallel course to that of the male group.

With PWM, the male over female increased production of IL6 was significant, whereas the similar trend in the Turner group did not reach statistical significance.

For TNFα, the LPS stimulation was not different between the three groups, presenting similar responses and LPS reactivity. Conversely, the PWM response, which occurred in a lower range than with LPS, shows an increased response in Turner compared to females.

The higher response of males to low stimulation with LPS is confirmed by the production of PGE-2.

Cytokine production levels in individuals are widely distributed, requiring relatively extended series of observations to obtain statistical significance in intergroup comparisons. This could be related to variations of cellular composition (mononucleated cells, neutrophils), although cultures with separated mononuclear cells display a larger coefficient of variation (reviewed in 19) that is attributed to non avoidable activation during the separation process.

In this study we observed that, in males and females, TNFα is highly correlated to IL6, provided low doses of LPS or PWM inducing responses in approximately the same relative range are used. This strong relationship is also visible with IL1, but only in the male group. Considering the potential anti-inflammatory function of IL6 [36], this relationship could reflect a regulation of these two cytokine levels by concomitant IL6 production. In this hypothesis, it is interesting to note that males have a significantly lower coefficient of variation (slope) than females for this relationship, as if the suggested influence of IL6 in reducing IL1 or TNFα production were higher in males. Caution must be taken, as the inter-individual variations are assimilated to potential intra-individual modulation in this model, a non demonstrated condition.

The Turner group tends to dissociate from the female group, expressing a lower LPS reactivity for IL1 responses and a dose response relationship equivalent to that of males for IL6, with a lowered LPS threshold level for maximal stimulation. For PWM stimulation, the Turner group follows more closely the male variations. Again, in the zone of low stimulation, their responses are elevated compared to females. The Turner group therefore behaves more like the male group with increased sensitivity to low levels of stimulation.

The possibility that increased expression of LPS receptors by monocytes may explain the observed functional differences, has been supported in by results in some animal models [19], but not in others [20]. To our knowledge no such data are reported in humans.

If we consider the in vivo counterpart of this LPS reactivity, a low level of stimulation by proinflammatory substances is likely to occur more frequently than higher levels. Therefore, it could be inferred that males and Turner subjects should be more prone to mount an inflammatory response than females with the beginning of infection.

This is exactly opposed to the actual in vivo situation. The paradox must be explained by factors not included in the ex vivo experiments. Multiple possibilities exist as the participation of cells not present in the tested blood sample (e.g. endothelial cells). These are able to locally produce several mediators with potential regulatory function, as well as modulating the traffic of cells. Further investigations are necessary to investigate the in vitro/in vivo apparent discrepancies.

The similarities between male and Turner syndrome group are in favour of some difference in gene expression between monosomy and disomy for the X chromosome.

## Conclusions

Stimulations by LPS and PW mitogen of blood cells from male and female prepubescent children showed quantitative and qualitative differences in the cytokine responses that could explain gender differences in the inflammatory profile. Higher levels of cytokines in males could be explained by differences in the complexe relationhip between inflammatory mediators with a possible role of IL6 that could be more potent in males than in females. Further studies are needed to investigate the possible role of X chromosome genes that could explain the observed differences.

## Competing interests

The authors declare that they have no competing interests.

## Authors' contributions

GC and JD drafted the manuscript. FH, LH, SM, CH, FC, JD and NL participated in the design of the study. GC and JD conceived of the study, and participated in its design and coordination. All authors read and approved the final manuscript.
